# Redefining Neonatal Vitamin A Adequacy and Deficiency Based on Maternal Nutrition: A Cross‐Sectional Study in Chongqing, China

**DOI:** 10.1002/fsn3.4552

**Published:** 2024-10-23

**Authors:** Xiaobing Fan, Xi Lai, Jingkun Miao, Qixiong Chen, Jia Chen, Huan Liu

**Affiliations:** ^1^ Mianyang Key Laboratory of Anesthesia and Neuroregulation, Department of Anesthesiology Mianyang Central Hospital Mianyang China; ^2^ Department of Respiratory and Critical Care Medicine, Mianyang Central Hospital, School of Medicine University of Electronic Science and Technology of China Mianyang China; ^3^ Department of Child Health Care, Guangzhou Women and Children's Medical Center Guangzhou Medical University, Guangdong Provincial Clinical Research Center for Child Health Guangzhou China; ^4^ Neonatal Screening Center Chongqing Health Center for Women and Children Chongqing China; ^5^ Department of Pediatrics Chongqing Traditional Chinese Medicine Hospital Chongqing China; ^6^ Department of Pediatrics, Mianyang Central Hospital, School of Medicine University of Electronic Science and Technology of China Mianyang China

**Keywords:** China, cross‐sectional study, mother, mother–child relationship, neonate, prenatal nutrition, vitamin a

## Abstract

There are no established diagnostic criteria for neonatal vitamin A deficiency (VAD), and applying adult VAD criteria to neonates may overestimate the neonatal VAD rate. This study aimed to evaluate neonatal vitamin A (VA) status and redefine thresholds for neonatal VA adequacy and deficiency based on maternal VA nutrition. A cross‐sectional study involving 1901 mother–neonate pairs was conducted in Chongqing, China. VA nutritional status was assessed by measuring serum VA levels and dietary VA intake from the third trimester to birth. The VAD rates of maternal dietary intake and serum were 27.091% and 23.356%, respectively, while 88.8% of neonates had serum VA levels < 0.70 μmol/L, the threshold for adult VAD. Neonatal VA levels were significantly lower than maternal levels. All neonates were healthy, with no clinical signs of VAD. Neonatal VA levels correlated positively with maternal VA levels. The threshold for neonatal VA adequacy was estimated to be ≥ 0.489 (95%CI: 0.464–0.512) μmol/L when maternal VA nutrition was adequate, and the 2.5th percentile of VA levels among all neonates was 0.192 μmol/L, predicting neonatal VAD. The study concluded that neonatal VAD rates might be overestimated, as most neonates received adequate VA from their mothers. Maternal VA status is a reliable predictor of neonatal VA status due to their close relationship. This study offers a new perspective on prenatal nutrition for determining neonatal VA adequacy and deficiency thresholds and developing neonatal VA supplementation programs. Further research is needed.

## Graphical abstract

Most neonates received adequate vitamin A (VA) nutrition from their mothers in utero, suggesting that the neonatal VA deficiency (VAD) rate may be overestimated. Maternal VA nutritional status appears to be a reliable predictor of neonatal VA status due to their close relationship. This study offers a new perspective on prenatal nutrition for determining neonatal VA adequacy and deficiency thresholds and developing neonatal VA supplementation programs.

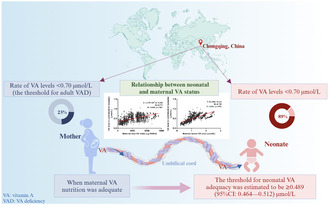

## 1 Introduction

Vitamin A (VA) is a fat‐soluble micronutrient essential for normal metabolism and function in the human body (Carazo et al. 2021; Hodge and Taylor 2022; Institute of Medicine (US) Panel on Micronutrients 2001). It plays crucial roles in cellular growth and differentiation, immune system function, bone and fetal development, and central nervous system formation (Carazo et al. 2021; Ortega et al. 1997; WHO 2009, 2016). Early in life, VA deficiency (VAD) can significantly impact growth and neurological development (de Souza Mesquita et al. 2021), while excessive VA intake poses a considerable risk of teratogenesis (Bastos Maia et al. 2019). Therefore, accurately determining VAD early in life is critical for developing effective nutritional intervention programs (Carazo et al. 2021). Otherwise, improper VA supplementation can lead to poisoning and teratogenesis (Carazo et al. 2021; Saad et al. 2021).

However, there are currently no diagnostic criteria for determining neonatal VAD (WHO 2009). Some studies have directly applied the World Health Organization (WHO) criteria for VAD in older children to neonates, considering serum VA levels of less than 0.70 μmol/L as deficient (Bezerra et al. 2020; Gomes et al. 2009; Hanson et al. 2018; WHO 2009). Studies using these criteria have reported neonatal VAD rates exceeding 60% (Bezerra et al. 2020; Hanson et al. 2018), suggesting that a majority of neonates may require therapeutic doses of VA supplements early in life, including at birth or even during pregnancy. Despite improvements in economic and nutritional status, VAD rates among older children and adults in many developing countries have significantly decreased (Stevens et al. 2015). It is noteworthy that even in developed countries, the rate of VAD in neonates remains remarkably high (Bezerra et al. 2020; Hanson et al. 2018). Therefore, it is imperative to explore the true nutritional status of neonatal VA and determine whether the VAD rate has been overestimated.

VA metabolism in neonates differs significantly from that in older children and adults, with VA reserves primarily dependent on placental transfer during late pregnancy (Azaïs‐Braesco and Pascal 2000). However, due to the limited window of placental transfer and the incomplete development of the fetal liver, the capacity to store VA in neonates is restricted (Hanson et al. 2016). The placenta acts as a selective barrier, regulating the passage of VA to prevent potential adverse effects of high concentrations on the fetus, which may contribute to the lower VA levels observed in newborns (Azaïs‐Braesco and Pascal 2000; Spiegler et al. 2012; Vinutha, Mehta, and Shanbag 2000; WHO 2009). Furthermore, neonatal VA metabolism is immature, with VA preferentially utilized for critical developmental processes such as the retina, lungs, and immune system, rather than maintaining high serum levels (Spiegler et al. 2012). Consequently, the lower serum VA concentrations at birth may reflect a normal physiological state rather than a deficiency in nutrition.

Our previous prospective longitudinal study found that the majority of healthy neonates with VA levels below 0.70 μmol/L at birth can attain normal levels by 6 months of age, even without additional VA supplementation (H. Liu et al. 2021). Throughout the follow‐up period, no neonates exhibited clinical VAD conditions (Gomes et al. 2009), and most demonstrated satisfactory growth and development (Liu et al. 2021). Neonatal VA levels naturally increase with age, and lower VA levels in neonates compared to older children may represent a normal physiological state (Liu et al. 2021). Consequently, applying VAD diagnostic criteria intended for older children to neonates may not be appropriate. It may be necessary to re‐examine the range of VA adequacy and deficiency in neonates to inform the development of a rational VA supplementation plan. Fetal VA is entirely derived from maternal sources (Hanson et al. 2016; Spiegler et al. 2012), and several small‐scale studies have also reported a close relationship between neonatal VA levels at birth and maternal VA levels (Bezerra et al. 2020; Chinyanga et al. 2005). Maternal VA status may therefore serve as a reliable predictor of neonatal VA adequacy or inadequacy at birth.

In this study, we conducted a comprehensive assessment of neonatal VA nutritional status at birth, as well as maternal dietary and serum VA status during pregnancy. Our aim was to demonstrate that the majority of neonates receive adequate VA nutrition from their mothers in utero and that the neonatal VAD rate may have been overestimated. Furthermore, our study confirmed a strong correlation between VA levels in neonates and their mothers within a large sample size, providing robust evidence for determining neonatal VA adequacy and deficiency thresholds and formulating neonatal VA supplementation programs from a novel perspective of prenatal nutrition.

## 2 Materials and Methods

### 2.1 Study Population, Setting, and Design

A population‐based, cross‐sectional study recruiting pregnant women and their neonates was conducted between March 2017 and January 2019 in Chongqing, China. This study was carried out in two representative large tertiary grade A hospitals in Chongqing, the Second Affiliated Hospital of Chongqing Medical University, in an urban area, and Qianjiang Central Hospital, in a suburban area. Chongqing is in southwest China and is one of China's most important central cities, with a total area of 82,400 km^2^, a permanent resident population of 31,243,200, and a birth population of 326,200 per year (Chongqing Municipal People's Government 2023; Chongqing Bureau of Statistics 2020).

Parturient women in these two hospitals from March 2017 to January 2019 who met the following inclusion criteria were enrolled in this study before delivery: (1) agreement to participate in this study; (2) singleton pregnancy; (3) prenatal examination showing no abnormality; and (4) no pregnancy complications, chronic diseases (including, but not limited to, severe or recurrent chronic anemia, diabetes, hypertension, nephropathy, epilepsy, or cancer), psychosis, or infectious diseases. The exclusion criteria for the mothers and neonates were as follows: (1) family disapproval; (2) inability to complete the dietary questionnaire; (3) no umbilical cord or maternal venous blood; (4) gestational age < 37 weeks or ≥ 42 weeks; (5) birth weight of neonates < 2500 or > 4000 g; or (6) neonates born with asphyxia, metabolic, or infectious diseases.

**FIGURE 1:**
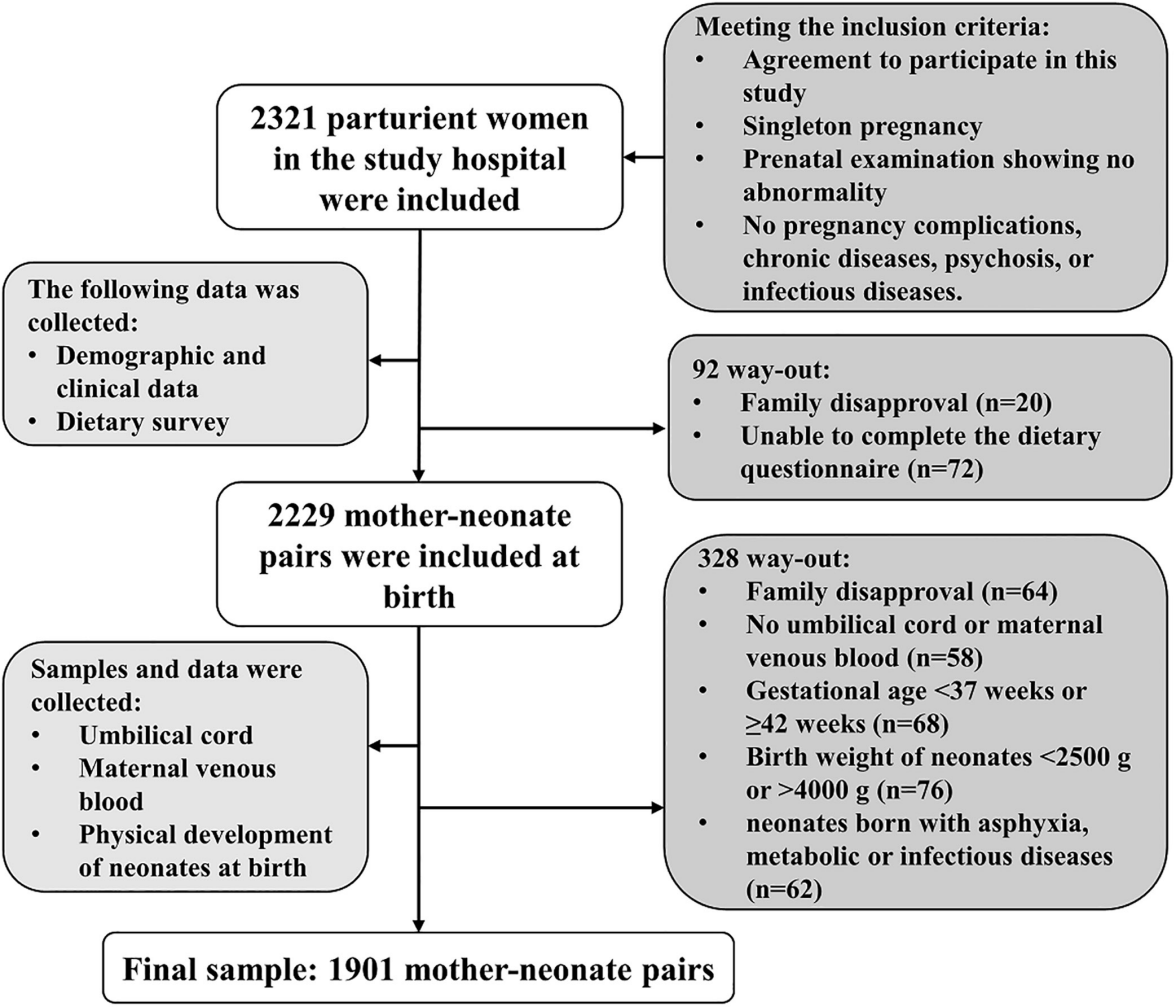
Sampling flowchart.

The following sample size calculation formula was used (Newcombe 1998): $n={\left(\frac{z_{1-\alpha /2}}{\delta }\right)}^{2}p\left(1-p\right)$, in which $z_{1-\alpha /2}$ = 1.96 (Newcombe 1998), and *δ*, the allowable error, was 3% (Newcombe 1998). *P* in the formula, the prevalence of neonatal VAD, is not clear due to the lack of VAD criteria for neonates. According to the formula, if *P* is 50% and the other variables are constant, the sample size will be the largest. It is also reported that *P* is assumed to be 50% when it is not clear (Newcombe 1998). Therefore, we assumed *P* of 50% in this study. The required maximum sample size was initially calculated as 1068. A 20% increase in sample size to account for sampling error (Bastos Maia et al. 2018) resulted in 1281. From March 2017 to January 2019, 3460 parturient women gave birth at these hospitals, and 2321 parturient women met the inclusion criteria and were included in this study before delivery. Following up after delivery, 420 (18.1%) families were excluded: 84 families (3.6%) withdrew their consent; 72 (3.1%) could not complete the dietary questionnaire; 58 (2.5%) had no umbilical cord or maternal venous blood; 68 (2.9%) had a gestational age < 37 weeks or ≥ 42 weeks; 76 neonates (3.3%) had a birth weight < 2500 or > 4000 g; and 62 neonates (2.7%) were born with asphyxia, metabolic, or infectious diseases. Ultimately, 1901 (81.9%) mother–neonate pairs were included in this longitudinal study at birth, well over the calculated 1281 (Figure 1).

All subjects gave informed consent for inclusion before participating in the study. The study was conducted in accordance with the Declaration of Helsinki, and the protocol was approved by the Medical Ethics Committee (due to the double‐blind review, the specific ethics committee institution and ethics number are indicated on the title page).

### 2.2 Study Procedures

#### 2.2.1 Clinical Data Collection

After obtaining informed consent, the history of pregnancy and delivery and demographic and clinical data, such as maternal educational level, maternal age, prepregnancy, body mass index (BMI), weight gain during pregnancy, parity, gestational age, and sex of neonates, were collected from the electronic medical records of the participants and the questionnaire. Neonate physical development at birth was monitored by measuring weight, length, and head circumference. Weight was measured to the nearest 10 g with an electronic baby scale (LEKA, HW‐B60). Length was measured using a measuring bed (LEKA, HW‐B60), and the head circumference was measured using a flexible ruler (Deli, 8213), both with a precision of 1 mm. The measured value was the average value after three repeated measurements.

#### 2.2.2 Dietary Data Collection

The food frequency questionnaire (FFQ) is a qualitative, quantitative, or semiquantitative dietary intake assessment method (Vijay et al. 2020). It has been widely used in large‐scale epidemiological studies due to its easy administration, low burden on participants and staff, and low cost compared to other assessment methods (Vijay et al. 2020). This study used the FFQ to assess dietary VA intake during the third trimester of pregnancy. The FFQ listed VA supplements and VA‐rich food (Hodge and Taylor 2022) commonly consumed by locals, such as carrots, pumpkins, liver, etc. FFQ investigators were trained clinical nutritionists. Prior to the implementation of the FFQ, participants were trained in portion estimation. They were shown food models with known weights and instructed to estimate the portion sizes of their daily food based on the food models. Participants reported the portion sizes and frequency of consumption of food items based on daily, weekly, and monthly intakes over the third trimester (the past 3 months). The frequency of intake for each food item on the FFQ was multiplied by the reported portion size and its respective VA content, which was derived from the Chinese Food Ingredients List (Chen et al. 2020). From this, the daily intake of VA (μg RAE/day) during the third trimester was calculated. Before the main study, local dietary habits were surveyed, and a pilot test of the FFQ was conducted with a small group of pregnant women. This ensured that vitamin A‐rich foods included in the questionnaire aligned with local dietary patterns, and that the questions were well designed, clear, and easy to answer. A subsample of 200 pregnant women with relatively stable diets was selected for repeated testing at two time points, spaced 2 weeks apart. The correlation coefficient for VA intake between the two tests exceeded 0.80, confirming the internal consistency of the FFQ. Additionally, a 24‐h dietary recall was used to assess VA intake over the past 3 days in this subset of 200 participants. The results showed no significant differences compared to the FFQ data, further validating the accuracy of the questionnaire.

#### 2.2.3 Blood Sample Collection

Samples of both cord and maternal blood were collected at delivery. One‐milliliter blood samples were collected, centrifuged, and stored at −80° within 12 h. The detection of retinol in blood was performed within 2 months.

#### 2.2.4 Measurement of Serum Retinol

VA levels in vivo are usually assessed by measuring serum retinol levels (Quadro 2016). Serum samples were tested at the Pediatric Research Institute, Children's Hospital of Chongqing Medical University. The serum retinol level was measured by high‐performance liquid chromatography–tandem mass spectrometry (HPLC–MS/MS) (API3200, AB SCIEX, 500 Old Connecticut Path Framingham, MA, USA) (Liu et al. 2021). Briefly, serum samples (20 μL) were deproteinized with methanol containing an internal standard (0.5 μg/mL d6‐retinyl acetate), extracted with hexane, evaporated to dryness under nitrogen, and reconstituted in methanol (Liu et al. 2021). Retinol in serum was separated by HPLC on a Shimadzu C18 75 mm × 2.0 mm column and quantitated by MS. All procedures were performed in a dark room to protect the samples from light. The lowest sensitivity of the measurement was 0.014 μmol/L for retinol.

#### 2.2.5 Definitions

Maternal age was categorized into three groups: < 25, 25–35, and ≥ 35 years old (Loy et al. 2019). Prepregnancy BMI was categorized into three groups: lean, < 18.5; normal, 18.5–23.9; overweight/obesity, ≥ 24 kg/m^2^ (Shen et al. 2019). Weight gain during pregnancy was categorized into three groups (inadequate, normal, and excessive) according to the Institute of Medicine (IOM) recommendations (Institute of Medicine (US) and National Research Council (US) Committee to Reexamine IOM Pregnancy Weight Guidelines 2009; Shen et al. 2019). According to Dietary Reference Intakes (DRIs) formulated by the Chinese Nutrition Society (Gannon, Jones, and Mehta 2020; Cheng 2014), maternal VA intake was divided into two levels: < 530 μg RAE/day, which indicated inadequate VA intake under the Estimated Average Requirement (EAR); and ≥ 530 μg RAE/day, which was adequate and met the EAR. According to the recommendation of the WHO (Liu et al. 2020; WHO 2009, 2016), the VA status of pregnant women was classified as follows: VAD, VA < 0.70 μmol/L; marginal VAD, VA 0.70–1.05 μmol/L; and adequate VA, VA ≥ 1.05 μmol/L. There are currently no criteria for grouping neonatal VA levels, and this study applied the above criteria to neonates in the initial analysis. Specialists evaluated clinical VAD based on conditions such as night blindness, conjunctival xerosis, Bitot's spot, and corneal xerosis (Gomes et al. 2009).

### 2.3 Statistical Analysis

Data were analyzed using SPSS 20.0 software, and figures were generated using GraphPad Prism 5.0 software. Normally distributed continuous variables are presented as the mean ± standard deviation (SD), while categorical variables are described by frequency and percentage (%). Neonatal VA levels were compared with maternal VA levels using paired *t*‐tests. The distribution of VA levels in neonates was compared to that in mothers using the chi‐square test, with the Bonferroni method applied for multiple comparisons. The effect of maternal VA status on neonatal VA levels was assessed by covariance analysis. Multivariate linear regression analysis was employed to investigate the relationship between maternal VA status and neonatal VA levels. In these models, neonatal VA was the dependent variable, while maternal dietary VA intake or serum VA levels and other relevant factors (covariates) were the independent variables. Covariates included gestational age, parity, maternal educational level (college and below vs. university and above), maternal age, weight gain, prepregnancy BMI, and neonatal sex (female vs. male). Stepwise regression was used for variable selection. The R‐squared (R^2^) value was employed to evaluate the model's explanatory power. Residual analysis and the variance inflation factor were used to assess multicollinearity, the linearity assumption, and the independence assumption of the model. Normality and heteroscedasticity tests were conducted on the residuals to ensure the validity of model assumptions. A bootstrap method with 1000 iterations was applied to generate robust confidence intervals (CI). The regression coefficients (β) and their 95% CIs were reported. A bilateral 95% reference interval for VA levels was defined as the 2.5th–97.5th percentile. The threshold for VA adequacy, distinguishing it from marginal deficiency, was predicted using either the 2.5th percentile of neonatal VA levels born to mothers with adequate VA or neonatal VA levels estimated through a multivariate regression model based on maternal VA adequacy. Additionally, the threshold for VA deficiency was predicted using the 2.5th percentile of VA levels across all 1901 healthy neonates. Statistical significance was set at *p* < 0.05.

## 3 Results

### 3.1 Sociodemographic and Obstetric Characteristics of Participants

A total of 1901 mother–neonate pairs were included in the study for analysis. The average age of the pregnant women was 27.041 ± 5.462 (SD) years, and more than half were between 25 and 35 years old (Table 1). Nearly two‐thirds of pregnant women had a university degree or above. The ratio of primiparous women to multiparous women was approximately 1:1.274. The average prepregnancy BMI of pregnant women was 21.208 ± 2.473 kg/m^2^, which was more than two‐thirds of the normal range. The average weight gain of pregnant women during pregnancy was 12.880 ± 4.057 kg, approximately 50% normal. The average gestational age was 39.550 ± 1.081 weeks. The ratio of females to males at birth was 1:1.190. The average weight of neonates at birth was 3322.790 ± 322.982 g, the average length was 49.947 ± 1.755 cm, and the average head circumference was 34.295 ± 1.367 cm.

**TABLE 1:** Sociodemographic and obstetric characteristics and VA intake of participants in Chongqing, Southwest China.

Characteristic	*n*	Mean ± SD or percentage
Maternal age (years)	1901	27.041 ± 5.462
< 25	635	33.403%
25–35	1087	56.865%
≥ 35	179	9.416%
Maternal educational level	1901	
College and below	726	38.190%
University and above	1175	61.810%
Parity (times)	1901	
0	836	43.977%
≥ 1	1065	56.023%
Prepregnancy BMI (kg/m^2^)	1901	21.208 ± 2.473
< 18.5	245	12.888%
18.5–23.9	1378	72.488%
≥ 24.0	278	14.624%
Weight gain during pregnancy (kg)	1901	12.880 ± 4.057
Inadequate	657	34.561%
Normal	945	49.711%
Excessive	299	15.729%
Gestational age (weeks)	1901	39.550 ± 1.081
Sex of neonates	1901	
Female	869	45.713%
Male	1034	54.392%
Neonatal weight (g)	1901	3322.790 ± 322.982
Neonatal length (cm)	1901	49.947 ± 1.755
Neonatal head circumference (cm)	1901	34.295 ± 1.367
Maternal total VA intake (μg RAE/day)	1901	1039.743 ± 598.217
< 530	515	27.091%
≥ 530	1386	72.909%
Proportion of dietary VA from food	1901	61.915%
Proportion of dietary VA from VA supplements	1901	38.085%
Maternal VA intake from food (μg RAE/day)	1901	470.148 ± 329.884
< 530	1252	65.860%
≥ 530	649	34.140%
Composition of dietary VA from food	1901	
Provitamin A carotenoids	1901	51.328%
Preformed VA	1901	48.672%
Maternal VA supplement (μg RAE/day)	1901	569.595 ± 595.077
0	963	50.658%
< 600	22	1.157%
< 1200	76	3.998%
≥ 1200	840	44.187%

Abbreviations: BMI, body mass index; SD, standard deviation; VA, vitamin A.

### 3.2 Dietary and Serum VA Status of Mothers and Neonates

The mean maternal VA intake was 1039.743 ± 598.217 μg RAE/day, and approximately a quarter of pregnant women (27.091%) had inadequate VA intake during the third trimester (Table 1). VA from supplements accounted for 38.085% of total VA intake, and nearly half of the mothers took VA supplements. Up to 65.860% of mothers who did not take VA supplements had inadequate VA intake. Further analysis of dietary structure revealed that the amount of preformed VA accounted for only half of the total VA intake from food.

The average maternal serum VA level was 1.062 ± 0.472 μmol/L, while the average VA level of neonatal cord blood was 0.491 ± 0.164 μmol/L. The VA level of neonates was significantly lower than that of mothers by paired *t* test (*p* < 0.001) (Table 2). The proportion of mothers with serum VA levels < 0.70 μmol/L was 23.356%. The proportion of neonates with cord blood VA levels < 0.70 μmol/L was 88.848%, which was significantly higher than that of mothers according to the chi‐square test (*p* < 0.05). In the included population, no subjects had clinical VAD conditions, such as night blindness, conjunctival xerosis, or Bitot's spot.

**TABLE 2:** VA levels in mothers and neonates and their comparison.

	Maternal serum (Mean ± SD or percentage (*n*))	Neonatal cord blood (Mean ± SD or percentage (*n*))	*t* or chi‐square	*p*
VA level (μmol/L)	1.062 ± 0.472 (1901)	0.491 ± 0.164 (1901)	67.271	< 0.001^a^
< 0.70	23.356% (444)	88.848%^b^ (1689)	1770.576	< 0.001^c^
0.70–1.05	30.931% (588)	11.099%^b^ (211)		
≥ 1.05	45.713% (869)	0.053%^b^ (1)		

Abbreviations: SD, standard deviation; VA, vitamin A.

^a^ Significant difference (*p* < 0.001) in VA level of neonates compared to mothers by paired *t* test.

^b^ Significant difference (*p* < 0.05) in VA level distribution (the percentage of VA level < 0.70, 0.70–1.05 and ≥ 1.05 μmol/L, respectively) of neonates compared to mothers by Bonferroni multiple comparison after chi‐square test.

^c^ Significant difference (*p* < 0.001) in VA level distribution of neonates compared to mothers by chi‐square test.

### 3.3 The Close Association of VA Nutritional Status Between Mothers and Neonates

**FIGURE 2:**
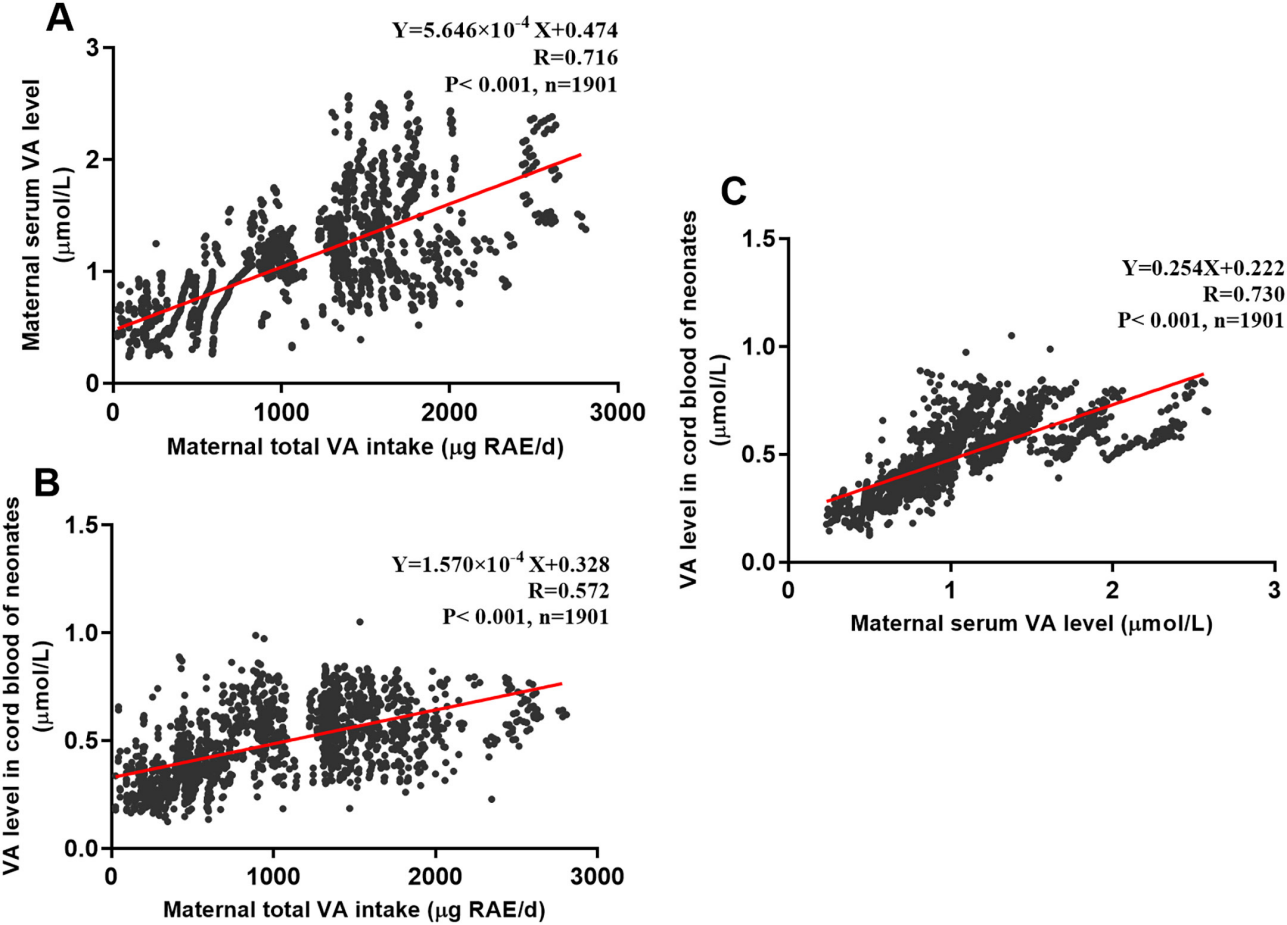
The relationship of VA level in neonates with maternal VA status. (A) Maternal serum VA level with maternal VA intake. (B) VA level in neonates with maternal VA intake. (C) VA level in neonates with maternal serum VA level. The results were obtained by multivariate linear regression analysis, adjusted for gestational age, parity, maternal educational level (college and below vs. university and above), maternal age, weight gain, prepregnancy BMI, and sex of neonates (female vs. male). Regression models were fitted by the stepwise method. These adjusted variables with no statistical significance were excluded from models and were not presented in the formulas. Abbreviations: BMI, body mass index; VA, vitamin A.

Next, we explored the association of VA nutritional status between mothers and neonates by multivariate linear regression models. All multivariate linear regression models were adjusted for gestational age, parity, maternal educational level (college and below vs. university and above), maternal age, weight gain, prepregnancy BMI, and sex of neonates (female vs. male). According to the regression analysis, maternal serum VA level (adjusted *R* = 0.716, *p* < 0.001) and cord blood VA level in neonates (adjusted *R* = 0.572, *p* < 0.001) were significantly positively correlated with maternal VA intake (Table 3 and Figure 2). The model predicted that the VA levels of mothers and neonates increased by 5.646 × 10^−4^ and 1.570 × 10^−4^ units, respectively, for every unit increase in maternal VA intake. In addition, the maternal VA level was a potentially positive predictor of neonatal VA (adjusted *R* = 0.730, *p* < 0.001). For every unit increase in maternal VA level, neonatal VA increased by 0.254 units.

**TABLE 3:** Multivariate linear regression analysis testing the relationship of VA status between mothers and neonates.

	Adjusted model^a^				
	*B*	CI (95%)	*p*	*F*	Adjusted *R*
Model 1^b^					
Maternal serum VA level^c^					
Constant	0.474	0.445–0.504	< 0.001	1992.158	0.716
Maternal VA intake^d^	5.646 × 10^−4^	(5.398–5.895) × 10^−4^	< 0.001		
Model 2^e^					
VA level in cord blood of neonates					
Constant	0.328	0.316–0.340	< 0.001	921.904	0.572
Maternal VA intake	1.570 × 10^−4^	(1.468–1.671) × 10^−4^	< 0.001		
Model 3^f^					
VA level in cord blood of neonates					
Constant	0.222	0.209–0.234	< 0.001	2172.376	0.730
Maternal VA level	0.254	0.243–0.265	< 0.001		

Abbreviations: VA, vitamin A; CI, confidence interval.

^a^ Adjusted for gestational age, parity, maternal educational level (college and below vs. university and above), maternal age, weight gain, prepregnancy BMI and sex of neonates (female vs. male). Regression models were fitted by the stepwise method. Adjusted variables with no statistical significance were excluded from models and their parameter estimates are not presented in Table 3.

^b^ Maternal serum VA level as the dependent variable, and constant and maternal VA intake as predictors in model 1.

^c^ VA level is measured in μmol/L.

^d^ VA intake is measured in μg RAE/day.

^e^ VA level in cord blood of neonates as the dependent variable, and constant and maternal VA intake as predictors in model 2.

^f^ VA level in cord blood of neonates as the dependent variable, and constant and maternal VA level as predictors in model 3.

Furthermore, we investigated the effect of different maternal VA statuses on neonatal VA levels by covariance analysis. The covariance analysis controlled for the effects of gestational age, parity, maternal educational level (college and below vs. university and above), maternal age, weight gain, prepregnancy BMI, and sex of neonates (female vs. male). The adequate VA status of mothers was associated with a significantly higher cord blood VA level in neonates (*F* = 2593.310, *p* < 0.001) (Table 4). Adequate VA status was defined as a maternal VA intake of ≥ 530 μg RAE/day and serum VA level of ≥ 1.05 μmol/L.

**TABLE 4:** Analysis of covariance testing the effect of maternal VA status on neonatal VA level.

	VA level in cord blood of neonates^a^				
	*n*	Mean ± SD	2.5th percentile^b^	*F* ^c^	*p*
Maternal VA status^d^					
Potentially inadequate	596	0.333 ± 0.114	—	2593.310	< 0.001
Adequate	868	0.621 ± 0.098	0.459		

Abbreviations: BMI, body mass index; SD, standard deviation; VA, vitamin A.

^a^ VA level is measured in μmol/L.

^b^ The 2.5th percentile indicating the lower bound of bilateral 95% reference interval.

^c^ The analysis of covariance controlling for the effects of gestational age, parity, maternal educational level (college and below vs. university and above), maternal age, weight gain, prepregnancy BMI and sex of infants (female vs. male).

^d^ Potentially inadequate VA status was defined as maternal VA intake < 530 μg RAE/day or serum VA level < 0.70 μmol/L; adequate VA status was defined as maternal VA intake ≥ 530 μg RAE/day and serum VA level ≥ 1.05 μmol/L.

### 3.4 The Possible Range of VA Adequacy and Deficiency in Neonates Based on Maternal Nutritional Status

The possible threshold of VA adequacy distinguished from marginal deficiency was predicted using the 2.5th percentile of VA levels of healthy neonates or the determination of VA levels of neonates from the above multivariate linear regression models, all of whom were born to mothers with adequate VA nutrition. Directly, the 2.5th percentile of cord blood VA levels in neonates whose mothers had adequate VA status was 0.459 μmol/L (Table 4). According to the above models, the neonatal VA level was ≥ 0.411 (95%CI: 0.394–0.429) μmol/L when the maternal VA intake was adequate (VA intake ≥ 530 μg RAE/day) (Figure 2B and Table 3). When the maternal serum VA level was adequate (VA level ≥ 1.05 μmol/L), the neonate VA level was ≥ 0.489 (95%CI: 0.464–0.512) μmol/L (Figure 2C and Table 3).

Then, the threshold of VA deficiency was predicted using the 2.5th percentile of VA levels of all 1901 healthy neonates, and it was 0.192 μmol/L.

## 4 Discussion

According to the WHO (2009), a serum VA level < 0.70 μmol/L is the diagnostic criterion for VAD in adults and older children aged 6–70 months. However, there are no diagnostic criteria for neonatal VAD, and many studies directly apply the criteria for older children to neonates (Bezerra et al. 2020; Hanson et al. 2018). Despite significant improvements in living standards and nutrition, reports indicate that the neonatal VAD rate remains high (Bezerra et al. 2020; Hanson et al. 2018). It is uncertain whether using the diagnostic criteria for older children overestimates the neonatal VAD rate (Bezerra et al. 2020). This study found that up to 88% of neonates had serum VA levels below 0.70 μmol/L, aligning with other reports (Bezerra et al. 2020; Hanson et al. 2018; Thoene et al. 2020). Our previous follow‐up study showed that the VA levels of most neonates with initial VA levels < 0.70 μmol/L increased to above 0.70 μmol/L by 6 months, even without intensive nutritional intervention (Liu et al. 2021). This suggests that low VA levels at birth may be a normal physiological state that improves with age (Liu et al. 2021).

In this study, we verified the above viewpoint from another perspective. VA, an essential nutrient, cannot be synthesized by the embryo and must be acquired from the maternal circulation through the placenta (Quadro and Spiegler 2020). We comprehensively evaluated maternal VA nutritional status using a large‐scale dietary survey. The study found that the rate of maternal VAD (serum VA level < 0.70 μmol/L) at birth was only 23%, significantly lower than that of neonates. Additionally, the dietary survey revealed that only 27% of mothers in the third trimester had insufficient VA intake, likely due to the widespread use of VA supplements, with approximately 50% of mothers taking them. Adequate VA levels in maternal diet and blood indicated that the sources of VA nutrition in utero were generally sufficient for neonates. Unlike other studies, the neonates included in this study were all healthy singletons, and risk factors affecting VA levels, such as preterm birth, multiple births, and infection, were excluded (Bezerra et al. 2020; Hanson et al. 2018). Furthermore, none of the subjects had clinical VAD conditions, such as night blindness, conjunctival xerosis, or Bitot's spots (Gomes et al. 2009). Therefore, there was no indication of widespread VA deficiency among the included neonates, and no clinical signs of VA deficiency were observed. Neonates with VA levels below 0.70 μmol/L may not necessarily be deficient in VA. This study and others have shown that neonatal VA levels were significantly lower than maternal VA levels, reaching only half of maternal VA levels (Baydas et al. 2002; Gomes et al. 2009; WHO 2016). As a unique group, the physiological VA levels of neonates may be much lower than those of older children and adults (Lindblad et al. 1998; Vinutha, Mehta, and Shanbag 2000; WHO 2016). Thus, the actual rate of VAD in neonates may not be as alarming as previously thought. The VAD diagnostic criteria for older children may overestimate the rate of neonatal VAD and are not applicable to neonates. This suggests that not all neonates below the adult VAD threshold require VA supplementation, potentially avoiding the adverse events associated with excessive VA supplementation.

To establish a basis for formulating a rational VA supplementation plan, it is essential to re‐evaluate the range of VA adequacy and deficiency in neonates. Since no de novo synthesis of VA occurs in the fetus, the fetus relies entirely on maternal circulating VA as the primary source of VA (Hanson et al. 2016; Spiegler et al. 2012). This indicates that neonates with adequate maternal VA nutrition are more likely to have normal VA levels. Previous small‐scale studies in Zimbabwe and Brazil have demonstrated a significant correlation between maternal and neonatal VA status at birth (Bezerra et al. 2020; Chinyanga et al. 2005). In this study, we expanded the sample size, comprehensively assessed maternal VA nutritional status through dietary surveys, and excluded common confounding factors affecting VA levels (Campos et al. 2019; Gebreselassie, Gase, and Deressa 2013; Song et al. 2017; WHO 2009). The results showed that neonatal VA levels were closely related to maternal VA nutritional status in late pregnancy, highlighting maternal VA status as a crucial factor influencing neonatal VA levels at birth. Thus, maternal VA status may be a reliable predictor of neonatal VA adequacy at birth. The relationship between maternal and neonatal VA levels at birth is a complex physiological process involving multiple mechanisms, including placental transport, maternal nutritional status, placental barrier function, and maternal‐fetal metabolic coordination (Gannon, Jones, and Mehta 2020; McCauley et al. 2015). However, these mechanisms are not yet fully elucidated. Future studies should focus on exploring the biological pathways of VA metabolism during pregnancy and neonatal life to provide a deeper understanding of the mother–neonate relationship and determinants of neonatal VA status.

To estimate neonatal VA status, we visualized the relationship between maternal and neonatal VA levels using multivariate linear regression models. According to these models, neonatal VA levels were ≥ 0.489 (95%CI: 0.464–0.512) μmol/L when maternal serum VA and dietary VA intake were adequate. Additionally, the 2.5th percentile of cord blood VA levels in neonates whose mothers had adequate VA status was 0.459 μmol/L. Furthermore, the 2.5th percentile of VA levels among all 1901 healthy neonates was 0.192 μmol/L. Based on these results, we predicted that the threshold for VA adequacy, distinguishing it from marginal deficiency, is 0.489 (95%CI: 0.464–0.512) μmol/L, and the threshold for VA deficiency in neonates is 0.192 μmol/L. Our previous study found that neonates with VA levels ≥ 0.588 μmol/L at birth could achieve levels greater than 1.05 μmol/L by 6 months of age, meeting the adequacy criteria for children aged 6–70 months (Liu et al. 2021; WHO 2009). The adequate VA range (≥ 0.588 μmol/L) predicted by postnatal VA nutrition aligns closely with that predicted by prenatal VA nutrition in this study. Utilizing a large sample and correcting for confounding factors, this study explored the potential thresholds for VA adequacy and deficiency in healthy neonates from a new prenatal nutrition perspective.

Our study preliminarily identified a new boundary point for adequate VA at 0.489 (95%CI: 0.464–0.512) μmol/L, which is significantly lower than the previously established 1.05 μmol/L. This finding suggests that for neonates with VA levels between 0.489 (95%CI: 0.464–0.512) μmol/L and 1.05 μmol/L, the previously recommended high therapeutic doses of VA may increase the risk of toxicity and impose an economic burden. Prophylactic doses of VA supplementation might be more appropriate for this group. For neonates with marginal VAD (0.192–0.489 μmol/L) and VAD (≤ 0.192 μmol/L), timely therapeutic doses of VA may be necessary. As the thresholds for neonatal VA adequacy and deficiency evolve, the prophylactic and therapeutic dosages may need adjustment.

Similarly, since VA levels in pregnant women are closely correlated with those in neonates, the dosage of VA supplementation in pregnant women might also need to be updated to reflect the different thresholds of VA adequacy and deficiency in neonates compared to adults. Insufficient or excessive prenatal VA nutrition could increase the risk of birth defects (Su et al. 2023). This study provides a basis for formulating neonatal VA supplementation programs by exploring new thresholds for neonatal VA adequacy and deficiency. Future research should aim to verify and refine these thresholds and explore prenatal and postnatal VA supplementation protocols for pregnant women and neonates based on the new cut‐point values.

Previous studies have suggested that the prevalence of neonatal VAD may be overestimated, but this hypothesis had not been experimentally tested (Bezerra et al. 2020; Hanson et al. 2018; Thoene et al. 2020). This study employed a large‐scale dietary survey to comprehensively assess maternal VA nutritional status during pregnancy, demonstrating from a new prenatal nutrition perspective that using older children's diagnostic criteria for VAD may overestimate the neonatal VAD rate. This finding aligns with the conclusions of our previous follow‐up study (Liu et al. 2021). Furthermore, based on the close relationship between maternal and neonatal VA levels, this study is the first to predict neonatal VA status using maternal VA levels during pregnancy and to explore the possible thresholds for neonatal VA adequacy and deficiency from a prenatal nutrition perspective. Compared to other studies (Bezerra et al. 2020; Hanson et al. 2018; Thoene et al. 2020), this study included a much larger sample size of healthy singleton neonates and excluded common confounding factors affecting VA levels, such as preterm birth, multiple births, and infection, making the conclusions more robust.

Considering the limitations of this study, including the single‐center design and potential recall bias from the retrospective approach, we recommend that future research involve multicenter prospective studies across different geographical locations to enhance the generalizability and representativeness of the results. Moreover, considering the dynamic changes in VA metabolism in neonates, prospective longitudinal studies should be conducted to track neonatal VA levels over time and validate the thresholds for adequacy and deficiency proposed in this study. For high‐risk groups such as preterm and multiple births, future research should explore whether different diagnostic criteria and supplementation protocols are necessary. Finally, future research should delve into the mechanisms underlying the relationship between maternal and neonatal VA levels to better understand neonatal VA metabolism and requirements.

In conclusion, most neonates received adequate VA nutrition from their mothers in utero, and the neonatal VAD rate has been overestimated. Maternal VA nutritional status is a reliable predictor of neonatal VA status due to their close relationship. This study provides a new perspective on prenatal nutrition for determining the thresholds for neonatal VA adequacy and deficiency and for developing neonatal VA supplementation programs. Further studies are needed to validate these findings.

## Author Contributions

H.L. and J.C. designed the research. H.L. and X.L. provided technical guidance and financial support for the study. X.F., X.L., H.L., J.C., J.M., and Q.C. conducted the research. X.F., X.L., and H.L. analyzed the data and wrote the paper. X.F. and X.L. contributed equally to this work. H.L. and J.C. had primary responsibility for final content. All authors read and approved the final manuscript. All authors agreed on the order in which their names were listed in the manuscript.

## Ethics Statement

This study was conducted in accordance with the Declaration of Helsinki, and the protocol was approved by the Medical Ethics Committee of Children's Hospital affiliated with Chongqing Medical University (022/2014).

## Consent

All subjects gave informed consent for inclusion before participating in this study.

## Conflicts of Interest

The authors declare no conflicts of interest.

## Data Availability

The corresponding author will provide the datasets used and analyzed during the current work upon reasonable request.
